# Foliar Spraying of NaHS Alleviates Cucumber Salt Stress by Maintaining N^+^/K^+^ Balance and Activating Salt Tolerance Signaling Pathways

**DOI:** 10.3390/plants12132450

**Published:** 2023-06-26

**Authors:** Shilei Luo, Zeci Liu, Zilong Wan, Xianxia He, Jian Lv, Jihua Yu, Guobin Zhang

**Affiliations:** 1 College of Horticulture, Gansu Agricultural University, Lanzhou 730070, China; luosl1021@163.com (S.L.); liuzc@gsau.edu.cn (Z.L.); wanzl@st.gsau.edu.cn (Z.W.); hxx_13359304716@126.com (X.H.); lvjian@gsau.edu.cn (J.L.); yujihuagg@163.com (J.Y.); 2 State Key Laboratory of Aridland Crop Science, Lanzhou 730070, China

**Keywords:** hydrogen sulfide, salt stress, ion transport, SOS, MAPK

## Abstract

Hydrogen sulfide (H_2_S) is involved in the regulation of plant salt stress as a potential signaling molecule. This work investigated the effect of H_2_S on cucumber growth, photosynthesis, antioxidation, ion balance, and other salt tolerance pathways. The plant height, stem diameter, leaf area and photosynthesis of cucumber seedlings were significantly inhibited by 50 mmol·L^−1^ NaCl. Moreover, NaCl treatment induced superoxide anion (O_2_^·−^) and Na^+^ accumulation and affected the absorption of other mineral ions. On the contrary, exogenous spraying of 200 μmol·L^−1^ sodium hydrosulfide (NaHS) maintained the growth of cucumber seedlings, increased photosynthesis, enhanced the ascorbate–glutathione cycle (AsA–GSH), and promoted the absorption of mineral ions under salt stress. Meanwhile, NaHS upregulated *SOS1*, *SOS2*, *SOS3*, *NHX1*, and *AKT1* genes to maintain Na^+^/K^+^ balance and increased the relative expression of *MAPK3*, *MAPK4*, *MAPK6*, and *MAPK9* genes to enhance salt tolerance. These positive effects of H_2_S could be reversed by 150 mmol·L^−1^ propargylglycine (PAG, a specific inhibitor of H_2_S biosynthesis). These results indicated that H_2_S could mitigate salt damage in cucumber, mainly by improving photosynthesis, enhancing the AsA–GSH cycle, reducing the Na^+^/K^+^ ratio, and inducing the SOS pathway and MAPK pathway.

## 1. Introduction

The major reason for increasing salt stress in agricultural soil is irrigation with poor saline water, poor drainage, the practice of irrigation, high transpiration, and low rainfall. High salt levels decrease the growth and yield of different crops in various ways [1,2,3]. Soil salinization is on the increase, and most of the cash crops are unable to adapt to this high-salt environment [4,5]. A high-salt environment inhibits growth, development, flowering and fruiting of crops, and restricts the water and nutrients uptake by crops, leading to the decline of yield quantity and quality [6]. The high concentration of salt ions causes the increase of soil osmotic potential, which leads to the inability of plants to absorb water. Osmotic stress leads to the generation of metabolic disorders such as membrane damage, reactive oxygen species (ROS) accumulation, and ion imbalance in plants [7,8]. Salt stress affects adversely on potassium and calcium by reducing K^+^ and Ca^2+^ ions absorption and transportation. Moreover, the accumulation of Na^+^ will cause mineral ion imbalance, nutrient deficiency symptoms, and metabolic disorders in plants, thus limiting plant growth and yield [9]. Furthermore, salt stress hinders growth by inhibiting different physiological and biochemical activities of plants, inhibiting photosynthesis, reducing nutrient absorption, and inhibiting cell division and expansion [10,11].

As a stationary organism, plants must formulate various strategies to adapt the salt environment, including signal transduction, ion transport and osmotic homeostasis. These mechanisms mostly depend on plant hormones, lipids, signal molecules, etc. As a new gas signal molecule, H_2_S has been reported to have a significant regulatory role in enhancing salt tolerance in plants [12,13]. In *Cyclocarya paliurus*, H_2_S alleviated the reduction of biomass, maintained chlorophyll fluorescence, and increased the activity of antioxidant enzymes, thus reducing the damage of salt stress [14]. Jing et al. found that salt stress increased endogenous H_2_S in cucumber, maintained the steady state of K^+^ and Na^+^, and inhibited the accumulation of ROS and membrane lipid peroxidation [15]. Liu et al. also studied the effect of NaHS application on salt tolerance in mangrove species, showing that H_2_S increased carbon fixation, chlorophyll content, and photosynthetic electron transfer rates. In addition, H_2_S could reduce oxidative damage by enhancing the AsA–GSH cycle and improving the activity of antioxidant enzymes [16]. H_2_S pretreatment enhanced salt resistance in wheat by reducing Na^+^ accumulation, decreasing the Na^+^/K^+^ ratio, increasing the rate of Na^+^ efflux, and increasing the selective transport of sodium by K^+^ [17]. The comparative proteomic analysis also showed that H_2_S was involved in alleviating oxidative damage, improving photosynthetic capacity, enhancing energy metabolism, and maintaining cell structure in rice seedlings under salt stress [18].

Na^+^ and K^+^ transporters are one of the mechanisms for plants to resist salt stress. The high-affinity potassium transporter (HKT) is a kind of membrane protein with ion transport characteristics; HKT1 can specifically transport Na^+^, maintain the balance of Na^+^/K^+^ in tissues and cells through long-distance transport and distribution of Na^+^. The expression of *HKT1* in this region can enable Na^+^ to be transported from xylem to xylem parenchyma cells, preventing Na^+^ from being transported to aboveground parts, thus ensuring that the photosynthesis of plants is not damaged [19,20]. NHX protein (Na^+^/H^+^ antiporters) is an important transporter for ion regionalization, and overexpression of *Arabidopsis* NHX1 (*AtNHX1*) in transgenic mung bean plants effectively segregates Na^+^ into vesicles and enhances salt tolerance [21]. A calcium-dependent protein kinase pathway of the salt overly sensitive (SOS) pathway mediates salt stress signal and Na^+^ tolerance, which is an important way for plants to resist salt stress. SOS1 is a main determinant of Na^+^ transport from the cytoplasm to the plasma ectodomain, driven by H^+^-ATPase at the plasma membrane [22]. SOS2 plays a central role in the SOS signaling pathway, and SOS2 can be activated by Na^+^ [23]. SOS3 and SCaBP8 (SOS3-LIKE CALCIUM-BINDING PROTEIN8) bind Ca^2+^ and interact with SOS2, enhancing SOS2 activity and bringing it to the plasma membrane, activating SOS1, and promoting Na^+^ efflux [24].

The mitogen-activated protein kinase (MAPK/MPK) cascade response pathway is a critical pathway to respond to adversity stress in plants, including salt, drought, cold, heat, and pathogenic bacteria [25,26]. Many reports suggested that MAPK was involved in the regulation of salt stress in plants. In *Arabidopsis*, MPK6 activates and interacts with MYB41 to increase salt tolerance [27]. The genes of *OsMPK44*, *OsMPK5,* and *OsMPK4* have also been shown to be associated with salt stress resistance in rice. Rice transgenic strains overexpressing *OsMPK5* and *OsMPK44* exhibited higher salt tolerance [28]. The expression levels of *TaMPK4*, *TaMPK6,* and *TaMPK17* in wheat gradually increased under high-salt treatment [29]. Therefore, this experiment aimed to investigate the regulatory role of H_2_S on physiological and biochemical mechanisms related to cucumber under salt stress, especially the effects of H_2_S on antioxidant systems, ion homeostasis, and salt tolerance signaling pathways.

## 2. Results

### 2.1. Exogenous NaHS Enhanced Cucumber Seedlings Growth under Salt Stress

**Figure 1:**
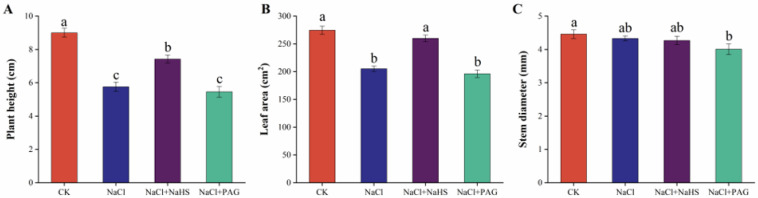
Effects of exogenous NaHS on plant height, stem diameter, and leaf area of cucumber seedlings under salt stress. The plant height (**A**), stem diameter (**B**), and leaf area (**C**) were measured after 7 d under different treatments. The values are mean ± SE of three independent experiments (n = 9). Different letters express significant differences by Duncan’s multiple range test (*p* < 0.05).

To evaluate the effect of exogenous NaHS on the growth of cucumber seedlings under salt treatment, we measured the plant height, stem diameter, and leaf area. Compared with CK, salt stress significantly decreased plant height and leaf area by 36.0% and 25.3%, respectively, and had no significant impact on stem diameter (Figure 1). The application of NaHS significantly alleviated the effects of salt damage and increased plant height and leaf area by 28.9% and 26.8%, respectively. However, The treatment of PAG (hydrogen sulfide synthesis inhibitor) + NaCl further deepened salt damage. Compared with salt treatment alone, plant height, stem diameter, and leaf area decreased by 5.21%, 7.39%, and 4.38%, respectively.

### 2.2. Exogenous NaHS Enhanced Photosynthesis of Cucumber Seedlings under Salt Stress

**Figure 2:**
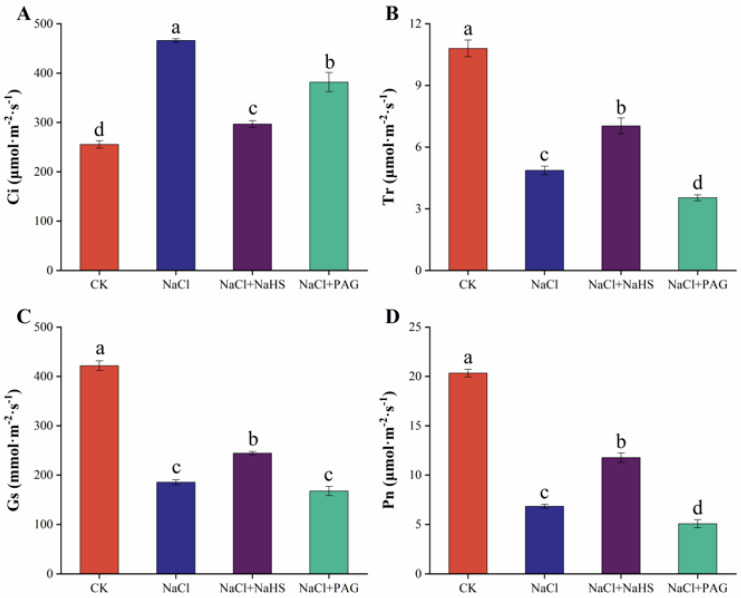
Effects of exogenous NaHS on photosynthesis of cucumber seedlings under salt stress. CK: distilled water; NaCl: 50 mmol·L ^−1^; NaCl + NaHS: 50 mmol·L ^−1^ + 200 μmol·L ^−1^; NaCl + PAG: 50 mmol·L ^−1^ NaCl + 150 μmol·L ^−1^ PAG. Determination of Ci (**A**), Tr (**B**), Gs (**C**), and Pn (**D**) after 7 d. The values are mean ± SE of three independent experiments (n = 3). Different letters express significant differences by Duncan’s multiple range test (*p* < 0.05).

In order to explore the effect of NaHS on photosynthesis, we measured the relevant parameters. As shown in Figure 2, compared with the CK, Tr, Gs, and Pn under NaCl treatment decreased by 54.9%, 56.0%, and 66.4%, respectively, while Ci increased by 82.5%. NaCl + NaHS treatment resulted in 44.5%, 31.8%, and 72.2% greater Tr, Gs, and Pn, respectively (Figure 2B–D), while decreased Ci by 36.3% (Figure 2A), compared to that of NaCl treatment. The influence of salt stress alone was exacerbated by PAG + NaCl treatment, resulting in a significant decrease of Pn by 75.1% compared to normal plants (Figure 2D).

### 2.3. Exogenous NaHS Maintains Chlorophyll Fluorescence of Cucumber Seedlings under Salt Stress

**Figure 3:**
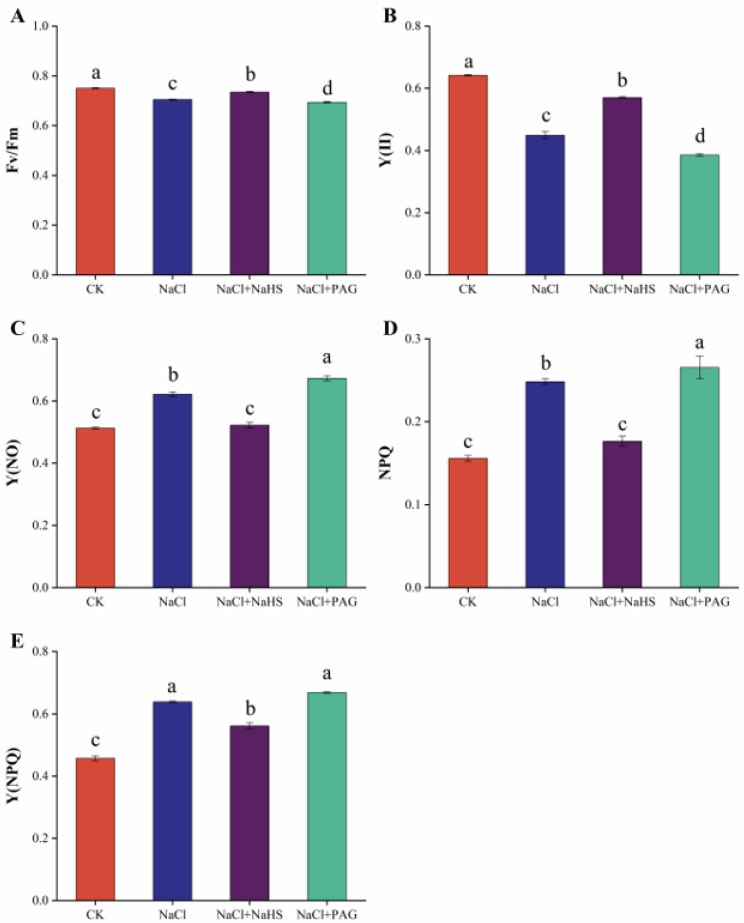
Changes in chlorophyll fluorescence parameters. Chlorophyll fluorescence parameters include Fv/Fm (**A**), Y(Ⅱ) (**B**), Y(NO) (**C**), NPQ (**D**), and Y(NPQ) (**E**). CK: distilled water; NaCl: 50 mmol·L ^−1^; NaCl + NaHS: 50 mmol·L ^−1^ + 200 μmol·L ^−1^; NaCl + PAG: 50 mmol·L ^−1^ NaCl + 150 μmol·L ^−1^ PAG. Determination of chlorophyll fluorescence parameters after 7 d. The values are mean ± SE of three independent experiments (n = 3). Different letters express significant differences by Duncan’s multiple range test (*p* < 0.05).

Chlorophyll fluorescence parameters are important indicators used to reflect the photosynthetic mechanism and photosynthetic physiology of plants and are internal parameters for studying the relationship between plant photosynthesis and the environment. As shown in Figure 3, The Fv/Fm and Y(II) of cucumber seedling leaves under NaCl treatment decreased significantly, while the NaCl + NaHS treatment maintained a higher quantum efficiency of PSII under salt stress, which was significantly higher than that of NaCl treatment. NaCl + PAG treatment aggravated the decrease of Fv/Fm and Y(II) under salt stress (Figure 3A,B). Compared with CK, NaCl treatment significantly increased the values of Y(NO), NPQ, and Y(NPQ). However, NaCl + NaHS treatment significantly inhibited the increase of Y(NO) NPQ and Y(NPQ) to reduce energy dissipation (Figure 3C–E).

### 2.4. Exogenous NaHS Inhibited the Accumulation of O_2_^·−^ in Cucumber Seedlings Induced by Salt Stress

**Figure 4:**
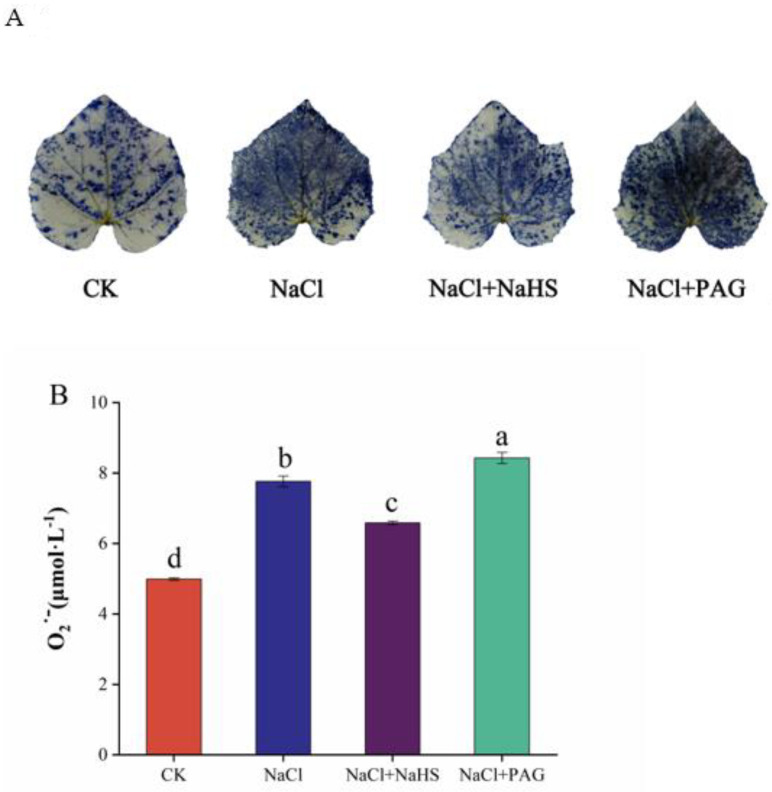
Effect of exogenous NaHS on O_2_^·−^ content in cucumber leaves. NBT staining was suitable for the qualitative determination of O_2_^·−^ in leaves (**A**). UV spectrophotometry was used for the quantitative determination of O_2_^·−^ (**B**). CK: distilled water; NaCl: 50 mmol·L ^−1^; NaCl + NaHS: 50 mmol·L ^−1^ + 200 μmol·L ^−1^; NaCl + PAG: 50 mmol·L ^−1^ NaCl + 150 μmol·L ^−1^ PAG. The values are mean ± SE of three independent experiments (n = 3). Different letters express significant differences by Duncan’s multiple range test (*p* < 0.05).

NBT staining results showed that the control plants had a small amount of O_2_^·−^ accumulation, and most of the leaves were transparent. Under NaCl treatment, the staining area of the leaves increased compared with CK; NaCl + NaHS treatment reduced the dyeing area of cucumber leaves under salt stress, while NaCl + PAG treatment deepened the dyeing degree (Figure 4A).

In order to verify the inhibition of H_2_S on the accumulation of O_2_^·−^, we measured the content of O_2_^·−^ in leaves. NaCl treatment alone and NaCl + PAG treatment significantly increased the value of O_2_^·−^ by 55.6% and 69.0% compared to the CK. H_2_S application significantly inhibited the accumulation of O_2_^−^ by 15.1% and 21.9% compared to salt stress and NaCl + PAG treatment (Figure 4B).

### 2.5. Exogenous NaHS Maintained Redox Balance by Enhancing AsA–GSH Cycle

**Figure 5:**
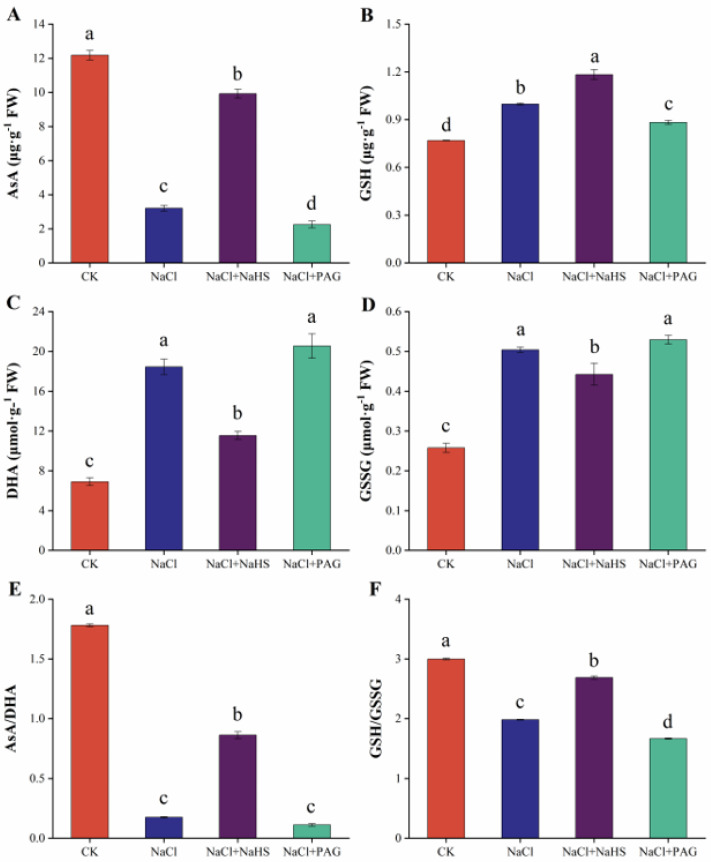
Effects of exogenous NaHS on AsA, GSH, DHA, GSSG, AsA/DHA, and GSH/GSSG of cucumber seedlings under salt stress. CK: distilled water; NaCl: 50 mmol·L ^−1^; NaCl + NaHS: 50 mmol·L ^−1^ + 200 μmol·L ^−1^; NaCl + PAG: 50 mmol·L ^−1^ NaCl + 150 μmol·L ^−1^ PAG. Determination of AsA (**A**), GSH (**B**), DHA (**C**), GSSG (**D**), AsA/DHA (**E**), and GSH/GSSG (**F**) after 7 d. The values are mean ± SE of three independent experiments (n = 3). Different letters express significant differences by Duncan’s multiple range test (*p* < 0.05).

AsA–GSH is a system for plants to scavenge free radicals and prevent membrane lipid peroxidation. We determined the members of the AsA–GSH cycle in cucumber leaves under salt stress. Compared with the CK, the content of AsA in cucumber leaves was significantly decreased, and the content of DHA was dramatically increased under salt stress, while under NaCl + NaHS treatment, the content of AsA and DHA was close to the CK level. Compared with salt stress, NaCl + PAG treatment significantly reduced the content of AsA but did not significantly change the content of DHA (Figure 5A,C). The AsA/DHA ratio of NaCl treatment was significantly lower than that of the control. NaCl + NaHS treatment significantly reduced the AsA/DHA ratio treated with salt, and there was no significant difference between NaCl + PAG treatment and NaCl treatment (Figure 5E).

Compared with the CK, salt treatment induced a significant increase in GSH content and GSSG content, while the GSH content in NaCl + NaHS treatment was significantly higher than that in NaCl treatment, and the GSSG content was significantly lower, but higher than that in the CK (Figure 5B,D). Under NaCl + PAG treatment, GSSG content had no significant difference compared with NaCl treatment, but GSH content decreased significantly (Figure 5B,D). Compared with NaCl treatment, the GSH/GSSG ratio under NaCl + NaHS treatment increased significantly, close to the control level, while the GSH/GSSG ratio under NaCl+PAG treatment decreased significantly (Figure 5F).

### 2.6. Exogenous NaHS Maintained Na^+^/K^+^ Balance of Cucumber Seedlings under Salt Stress

**Figure 6:**
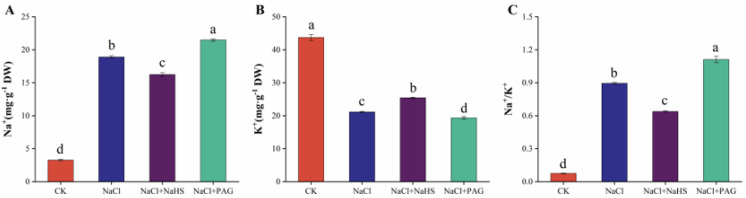
Effects of exogenous NaHS on Na^+^ and K^+^ contents in cucumber leaves under salt stress. CK: distilled water; NaCl: 50 mmol·L ^−1^; NaCl + NaHS: 50 mmol·L ^−1^ + 200 μmol·L ^−1^; NaCl + PAG: 50 mmol·L ^−1^ NaCl + 150 μmol·L ^−1^ PAG. Determination of the content of Na^+^ (**A**), K^+^ (**B**), and the ratio of Na^+^/K^+^ (**C**) after 7 d. The values are mean ± SE of three independent experiments (n = 3). Different letters express significant differences by Duncan’s multiple range test (*p* < 0.05).

The balance of Na^+^ and K^+^ ions is the key to the survival of plants under salt stress, so we measured the content of Na+ and K^+^ in cucumber leaves. Compared with CK, salt stress induced a significant accumulation of Na^+^, a significant loss of K^+^, and dramatically increased Na^+^/K^+^ (Figure 6). However, NaCl + NaHS treatment significantly inhibited the accumulation of Na^+^ content, which decreased by 14.1% compared with salt stress alone, and slowed down the loss of K^+^ content by 20.3%, reducing the Na^+^/K^+^ ratio by 28.6%. Compared with salt treatment alone, after NaCl + PAG treatment, Na^+^ content increased by 13.7%, K^+^ content decreased by 8.4%, the Na^+^/K^+^ ratio increased by 24.2%, and salt accumulation was more obvious.

### 2.7. Exogenous NaHS Alleviated the Inhibition of Cucumber Seedlings on the Absorption of Mineral Elements under Salt Stress

**Figure 7:**
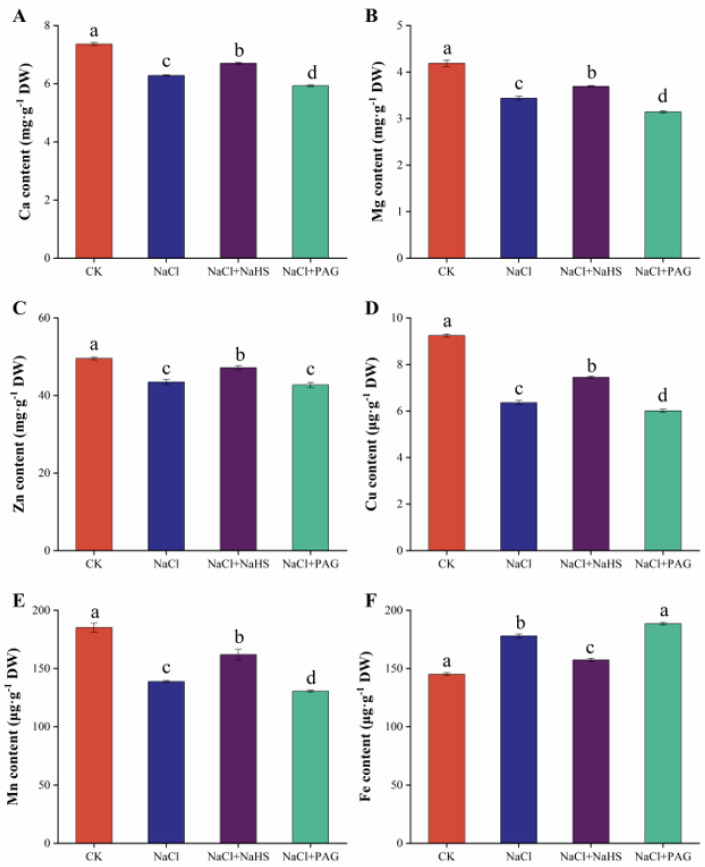
Changes in mineral elements content under different treatments. CK: distilled water; NaCl: 50 mmol·L ^−1^; NaCl + NaHS: 50 mmol·L ^−1^ + 200 μmol·L ^−1^; NaCl + PAG: 50 mmol·L ^−1^ NaCl + 150 μmol·L ^−1^ PAG. Determination of the content of Ca (**A**), Mg (**B**), Zn (**C**), Cu (**D**), Mn (**E**), and Fe (**F**) after 7 d. The values are mean ± SE of three independent experiments (n = 3). Different letters express significant differences by Duncan’s multiple range test (*p* < 0.05).

To investigate the influence of H_2_S on the uptake of mineral ions by cucumber in a high-salt environment, we analyzed the content of Ca, Mg, Zn, Cu, Mn, and Fe (Figure 7). The content of Ca, Mg, Mn, Cu, and Zn in plant leaves under salt stress were significantly decreased compared to CK, while Fe contents were increased. Ca, Mg, Mn, Cu, and Zn contents under NaCl + NaHS treatment were significantly higher compared to NaCl treatment. On the contrary, NaCl + PAG treatment disturbed the mineral element homeostasis of leaves, and the decrease in leaf Ca, Mg, and Cu contents under NaCl + PAG treatment was significantly higher than that under NaCl treatment, the increase in Fe content was significantly greater than that of NaCl treatment, and the decrease in Mn and Zn did not change significantly but was significantly lower than that of CK.

### 2.8. Exogenous NaHS Upregulated SOS Pathway Genes to Improve Cucumber Salt Resistance

**Figure 8:**
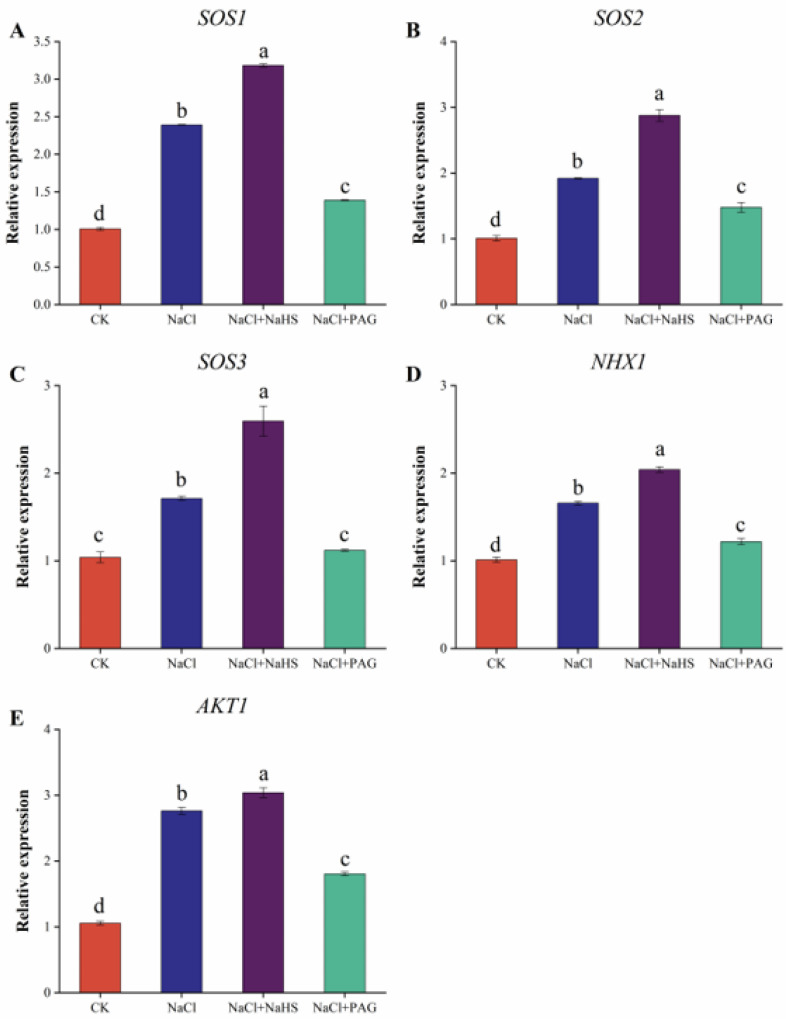
Effects of exogenous NaHS on Na^+^ and K^+^ transport-related genes in cucumber under salt stress. The relative expressions of SOS1 (**A**), SOS2 (**B**), SOS3 (**C**), NHX1 (**D**), and AKT1 (**E**) were measured after 7 d under different treatments. CK: distilled water; NaCl: 50 mmol·L^−1^; NaCl + NaHS: 50 mmol·L^−1^ + 200 μmol·L^−1^; NaCl + PAG: 50 mmol·L^−1^ NaCl + 150 μmol·L^−1^ PAG. The values are mean ± SE of three independent experiments (n = 3). Different letters express significant differences by Duncan’s multiple range test (*p* < 0.05).

To investigate the influence of H_2_S on Na^+^ and K^+^ transport genes, the relative expression of NHX1, AKT1, and SOS pathway-related genes was measured. As shown in Figure 8A–C, NaCl treatment significantly upregulated the relative expression of SOS1, SOS2, and SOS3 compared with CK. The relative expression of NaCl + NaHS treatment was 33.1%, 49.8%, and 51.5% markedly higher than these of salt treatment. Similarly, exogenous H2S further increased the expression of NHX1 and AKT1 genes by 23.0% and 10.0%, respectively, compared with salt alone (Figure 8D,E). However, NaCl + PAG treatment significantly downregulated the expression of SOS1, SOS2, SOS3, NHX1, and AKT1 genes by 41.9%, 23.0%, 34.5%, 26.6%, and 34.6% compared with salt treatment (Figure 8A–E). These results suggested that H_2_S upregulated Na^+^ and K^+^ transporter genes, while PAG administration inhibited this positive regulation.

### 2.9. Exogenous NaHS Enhanced Salt Tolerance of Cucumber by Upregulating MAPK Pathway-Related Genes

**Figure 9:**
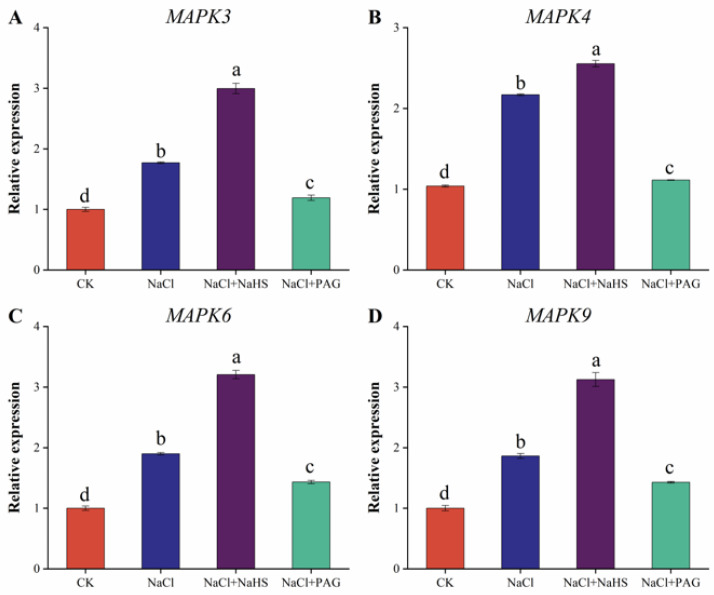
Effects of exogenous NaHS on MAPK pathway-related genes in cucumber under salt stress. The relative expressions of MAPK3 (**A**), MAPK4 (**B**), MAPK6 (**C**), and MAPK9 (**D**) were measured after 7 d under different treatments. CK: distilled water; NaCl: 50 mmol·L^−1^; NaCl + NaHS: 50 mmol·L^−1^ + 200 μmol·L^−1^; NaCl + PAG: 50 mmol·L^−1^ NaCl + 150 μmol·L^−1^ PAG. The values are mean ± SE of three independent experiments (n = 3). Different letters express significant differences by Duncan’s multiple range test (*p* < 0.05).

The MAPK pathway is one of the major ways for plants to resist abiotic stress, so we measured the relative expression of MAPK3, MAPK4, MAPK6, and MAPK9 genes (Figure 9). Compared with CK, salt treatment significantly upregulated the relative expression of MAPK3, MAPK4, MAPK6, and MAPK9 by 76.8%, 108.8%, 89.7%, and 85.9%, respectively. NaCl + NaHS treatment further upregulated the expression of MAPK3, MAPK4, MAPK6, and MAPK9, which were 69.1%, 17.7%, 68.7%, and higher than that of salt alone (67.8%). Conversely, NaCl + PAG treatment significantly inhibited the upregulation of MAPK3, MAPK4, MAPK6, and MAPK9 genes compared with NaCl treatment and NaCl + NaHS treatment. The above results showed that H_2_S resisted salt stress by upregulating the expression of MAPK pathway genes, while PAG (hydrogen sulfide synthesis inhibitor) could reverse this effect and weaken the ability of cucumber to resist salt stress.

## 3. Discussion

Salt toxicity is one of the main causes of crop yield limitation in arid regions, and usually salt stress inhibits crop growth and development [30,31]. The results of this study showed that salt stress significantly inhibited the plant height, stem diameter, and leaf area of cucumber seedlings. Exogenous H_2_S alleviated this negative effect and maintained the growth of cucumber seedlings, while PAG could partially reverse the alleviated growth inhibition (Figure 1). Similarly, treatments with concentrations of 50 and 100 µM NaHS resulted in a remarkable increase in the plant height, dry weight, and fresh weight of common bean under salt stress [32]. In wheat, H_2_S significantly improved various growth indicators of wheat and alleviated the inhibition of salt stress. Hypotaurine (HT, H_2_S scavenger) attenuated the positive effect of H_2_S on growth [17]. Salt stress inhibited the root length, stem length, fresh weight, and dry weight of *Brassica juncea*. However, H_2_S treatment significantly increased the plant growth parameters under stress conditions [33]. The above results indicated that exogenous H_2_S could maintain the growth of cucumber under salt stress.

Photosynthesis provides the material and energy basis for plant growth, but salt stress disrupts this action, resulting in crop growth and yield limitation. Salt stress can affect photosynthesis through stomatal limitation, resulting in reduced carbon assimilation [34], and also negatively affect the photosynthetic rate, osmotic potential, transpiration rate, and the relative water content and chlorophyll structure of plant leaves [35,36]. Our experiment showed that salt stress restricted Gs and Tr, while Ci increased, leading to a significant decrease in Pn (Figure 2). This result was consistent with Yan et al. and Sun et al. [1,37]. This study also found that H_2_S could maintain photosynthesis in cucumber seedlings and improve the net photosynthetic rate and stomatal conductance under salt stress (Figure 2C,D). Ding et al. (2019) reported that H_2_S pretreatment alleviated the reduction of Pn, Tr, and Gs in wheat seedlings caused by salt stress [38]. Salt stress significantly inhibited Gs, Pn, Ci, transpiration rate Tr, and chlorophyll properties in eggplant, while these photosynthetic parameters were reduced to a much lesser extent under H_2_S treatment [39]. In cucumber, exogenous H_2_S foliar treatment significantly improved photosynthetic parameters regardless of how salt stress limited photosynthesis [40]. Chlorophyll fluorescence analysis is a reference technique for studying information on the PSII status of plants [41]. Under salt treatment, photochemical burst parameters (Fv/Fm, Y(II), qP) and electron transport rate (ETR) were generally reduced, but nonphotochemical burst parameters (qN, NPQ, Y(NPQ)) were increased [35,42]. Our results were also similar; the value of Fv/Fm and Y(II) was significantly decreased under salt treatment compared with the normal plants (Figure 3A,B), while NPQ and Y(NPQ) were increased (Figure 3D,E). NaHS treatment maintained the photochemical efficiency and suppressed the dissipation of light energy (Figure 3). PAG treatment further deepened the damage of salt stress and reduced the value of Fv/Fm and Y(II). Similarly, in *Cyclocarya paliurus*, salt stress altered chlorophyll fluorescence parameters (Fv/Fm, NPQ, and ETR) and produced oxidative stress, whereas H_2_S treatment maintained chlorophyll fluorescence and reduced biomass loss [14]. Moreover, melatonin maintained chlorophyll fluorescence by increasing the content of endogenous H_2_S under salt stress, an effect that could be reversed by HT and PAG [1]. Salt stress significantly reduced the value of Fv/Fm. Pretreatment with NaHS enabled the plants to maintain the maximum PSII efficiency at a similar level to the control, while PAG application reduced the effect of H_2_S [43].

In plants, ion toxicity and osmotic stress caused by salt stress can lead to metabolic imbalance and accumulation of ROS, which eventually trigger oxidative damage in plants [44]. Salt stress induces the accumulation of ROS in plant organelles, such as chloroplasts, mitochondria, and plastid extracellular bodies. Plant cells sense the accumulated ROS and scavenge them through corresponding regulatory mechanisms, causing downstream signaling responses [10,45]. Our results indicated that salt stress induced O_2_^·−^ accumulation in leaves (Figure 4A), whereas spraying NaHS significantly reduced the production of O_2_^·−^ and alleviated the oxidative damage (Figure 4A,B). It has been shown that H_2_S treatment reduced the oxidative damage caused by salt stress by increasing the antioxidant capacity of bermudagrass seedlings, thus improving the tolerance of seedlings to salt stress to some extent [46]. Shan et al. (2014) also found that H_2_S maintained the redox status of ascorbic acid and glutathione by upregulating their metabolism and improving their antioxidant capacity, which in turn enhanced salt tolerance in maize [47]. In rice and wheat, H_2_S exerted its antioxidant effect in salt-stressed seedlings by increasing AsA and GSH levels to maintain redox homeostasis [48,49]. Another study also found that with the increase of NaCl treatment concentration, the L-cysteine desulfhydras (L-CD) activity and endogenous H_2_S content of alfalfa seedlings were induced to increase, and H_2_S as an endogenous regulator effectively regulated antioxidant enzyme activity, increased the GSH/GSSG ratio, and enhanced AsA–GSH cycle metabolism [50]. This was consistent with our results; H_2_S maintained redox balance by increasing the levels of AsA and GSH and decreasing the levels of DHA and GSSG under salt stress (Figure 5).

Salt stress induces the accumulation of Na^+^ in plant cells, eventually reaching toxic levels and disrupting ionic homeostasis [45,51]. In our study, salt treatment alone led to Na^+^ accumulation and the ratio of Na^+^/K^+^ increase. In addition, spraying 200 μmol·L^−1^ NaHS on leaves significantly inhibited the accumulation of Na^+^ and the efflux of K^+^, maintaining the ratio of Na^+^/K^+^ (Figure 6). In barley, NaHS (50 μmol·L^−1^) maintained Na^+^/K^+^ balance by reducing net K^+^ efflux and decreasing Na^+^ content [52]. Similar results were reported in *Medicago sativa* [50], *Populus euphratica* [53], wheat [17], and beans [32]. Mineral elements are usually present in plant cells as ions and are components of the structural material of the cells. The mechanisms of action of salt stress include the excessive accumulation of Na^+^ and Cl^−^ and the restricted uptake of other mineral ions [54]. In our study, salt stress led to a decrease in the uptake of Ca, Mg, Zn, Cu, and Mn elements (Figure 7A–E) and significantly increased the uptake of Fe in cucumber seedlings (Figure 7F). In contrast, H_2_S spraying promoted the uptake of Ca, Mg, Zn, Cu, and Mn elements and inhibited the uptake of Fe. NaCl + PAG treatment increased the salt effect and reversed the effect of H_2_S (Figure 7). This was consistent with the results in rice, eggplant, and tomato [39,48,55].

When plants are in the high-salt environment, high concentrations of Na^+^ will cause ion toxicity. Plants mainly use the SOS signal pathway to discharge Na^+^ or separate Na^+^ into vacuoles to regulate ion balance [45]. In our study, the SOS signal pathway was activated under salt stress, and *SOS1*, *SOS2,* and *SOS3* genes were significantly upregulated. Compared with salt treatment alone, exogenous NaHS spraying further increased the expression of *SOS1*, *SOS2,* and *SOS3*, while PAG treatment decreased the expression of them (Figure 8A–C). Chen et al. (2015) found that exogenous H_2_S reduced the level of cytoplasmic Na^+^ in barley seedling roots by regulating the expression level of *HvSOS1* and enhanced salt tolerance [52]. Moreover, NaHS evoked the transcriptional levels of *HvSOS1* genes’ upregulation in barley seedlings [52] and regulated the SOS1 channel to enhance salt tolerance [17]. NHX1 is a Na^+^/H^+^ antiporter protein located on the vesicle membrane that transports Na^+^ and K^+^ to the vesicle via a proton gradient [56]. AKT1 is an important channel protein involved in K^+^ uptake and is involved in plant response to salt stress [57]. We measured the relative expression of *NHX1* and *AKT1* under salt stress. The results indicated that H_2_S could significantly upregulate the expression of *NHX1* and *AKT1*, which further indicated that H_2_S resisted salt stress by discharging Na+ and maintaining K^+^ (Figure 8D,E). These results were confirmed by the study of Chen et al. They found that the application of NaHS to hybrid *Brassica napus* under salt stress differentially enhanced the expression of *NHX1*, *SOS2,* and *AKT1*, but it was impaired by either HT or PAG. At the same time, the plant had higher K^+^ accumulation and a lower Na^+^/K^+^ ratio [58]. Similar results were reported in mangrove [59] and barley [52].

Multiple components of the MAPK cascade response participate in plant salt stress signaling responses [60]. The MPK4/MPK6 cascade was involved in the Arabidopsis salt stress signaling response, and the MKK9-MPK3/MPK6 cascade was involved in the regulation of ethylene and phycocyanin biosynthesis and played a role in the *Arabidopsis* salt stress response [44]. *MAPK3*, *MAPK4*, *MAPK6,* and *MAPK9* participated in the resistance to salt stress in a variety of crops [61,62]. This experiment showed that *MAPK3*, *MAPK4*, *MAPK6,* and *MAPK9* were significantly upregulated by salt stress. Exogenous spraying of NaHS further upregulated the transcriptional expression of *MAPK3*, *MAPK4*, *MAPK6,* and *MAPK9* genes. NaCl + PAG treatment inhibited the effect of H_2_S and downregulated the expression of these genes (Figure 9). In wheat, NaHS + NaCl treatment induced obvious increases in TaMPK4 expression levels by 12.5%, 14.3%, and 18.8% after 1, 3, and 5 d of growth under salt stress compared to salt stress, respectively [38]. In cucumber, NaHS increased *CsNMAPK* expression, while NaHS inhibitor (PAG) and scavenger (HT) decreased its expression, indicating that NaHS treatment regulated MAPK expression. The MAPK inhibitor PD98059 reversed the effect of NaHS treatment, suggesting that MAPK activation was required for the alleviating effect of NaHS [63]. In addition, salt stress treatment upregulated the expression of *MAPK3/4/6/9* compared to CK. Melatonin pretreatment further increased the expression of *MAPK3/4/6/9*. However, HT treatment inhibited the expression of these genes compared with MT + NaCl treatment. These results suggested that the H_2_S and MAPK cascades crosstalk with each other in MT-induced salt stress and that H_2_S was an upstream signaling molecule for the MAPK cascade [1]. The above results suggested that the H_2_S-mediated MAPK pathway enhanced salt resistance in cucumber.

## 4. Materials and Methods

The “Xinchun No. 4” cucumber (*Cucumis sativus* L.) variety was selected as the experimental material. The clean and full seeds were soaked in warm water at 55 °C for 15 min, then water was added to normal temperature and soaked for another 8 h. Finally, the seeds were transferred to the incubator for germination. After 7 d of germination, the cucumber seedlings were transplanted into plastic boxes with Hoagland nutrient solution and continued to be cultured at a temperature of 25 °C/18 °C, a light intensity of 20,000 lux, and a day/night of 14 h/10 h. When seedlings grew to two leaves and one heart, four treatments were set: CK (Nutrient solution), NaCl treatment (50 mmol·L^−1^ NaCl), NaHS + NaCl (Foliar spray of 200 μmol·L^−1^ NaHS + 50 mmol·L^−1^ NaCl), PAG (H_2_S synthesis inhibitor) + NaCl (Foliar spray of 150 μmol·L^−1^ PAG + 50 mmol·L^−1^ NaCl). All indices were measured after 7 d following the above treatment.

### 4.1. Growth Index

Plant height was measured from the base of the rootstock to the apical growth point of the seedling. Stem diameter was determined with vernier calipers, and leaf area was measured for all functional leaves of the plant using a root analysis system (WinRHIZO Pro LA2400, Regent Inc., Vancouver, Canada), scanning the leaves, and batch analyzing leaf area.

### 4.2. Measurement of Photosynthetic Parameters and Chlorophyll Fluorescence Characteristics

The photosynthetic parameters of cucumber seedlings were measured with a portable photosynthetic instrument (CIRAS-2, PP Systems, Amesbury, USA). Put the plants to be tested under light to make them fully adapt to the light environment. The measurement indices include net photosynthetic rate (Pn), intercellular CO_2_ concentration (Ci), stomatal conductance (Gs), and transpiration rate (Tr) of seedling leaves [64].

A modulated chlorophyll fluorescence imaging system (IMAPING-PAM, Walz Company, Nuremberg, Bavaria, Germany) detected chlorophyll fluorescence parameters in cucumber leaves. Before measurement, the plant to be measured is put in a dark place to adapt to the darkness for at least 30 min. The measured parameters include the maximum photochemical efficiency (Fv/Fm) of PSII, the actual photochemical efficiency Y(II) of PSII under light adaptation, the quantum yield Y(NPQ) of regulatory energy dissipation, the quantum yield Y(NO) of nonregulatory energy dissipation, and the nonphotochemical quenching coefficient NPQ [64].

### 4.3. Determination and Dyeing of Superoxide Anion (O_2_^−^)

Determination of superoxide anion content: 0.5 g of cucumber leaves were weighed and added to 2 mL of extraction buffer. The mixture was homogenized in an ice bath and centrifuged at 8000 r/min and 4 °C for 10 min. The supernatant was collected. Then, 1 mL of the supernatant was mixed with 0.5 mL of 50 mmol·L^−1^ phosphate buffer (pH 7.8) and 0.1 mL of 10 mmol·L^−1^ hydroxylamine hydrochloride solution. The mixture was shaken and kept at 25 °C for 20 min. After that, 1 mL of 58 mmol·L^−1^ para-aminobenzenesulfonic acid solution and 1 mL of 7 mmol·L^−1^ α-naphthylamine solution were added, mixed, and shaken for 30 min at 30 °C. Finally, an equal volume of chloroform was added to extract the pigments. The mixture was centrifuged at 10,000 r/min for 3 min, and the pink upper aqueous phase was collected. The absorbance value was measured at 530 nm, and the content was calculated using a standard curve [65].

Nitroblue tetrazolium (NBT) staining: Cucumber leaves were collected after different treatments and washed with ultrapure water. The leaves were then placed in a conical flask, and 1 g·L^−1^ NBT staining solution was added. The flask was subjected to vacuum for 0.5 h and kept in the dark for 2 h. The stained samples were then soaked in a bleaching solution (glycerol:lactic acid:ethanol = 1:1:3) and boiled for several minutes until decolorization. Finally, the samples were removed and photographed [66].

### 4.4. AsA–GSH Cycle Determination

The contents of reduced glutathione (GSH), oxidized glutathione (GSSG), reduced ascorbic acid (AsA), and oxidized ascorbic acid (DHA) were determined using the instructions of the kit (comin, Suzhou, China).

### 4.5. Measurement of Mineral Element Content

Determination of mineral element content: Cucumber seedlings were collected as samples and placed in a preheated oven at 105 °C for 15 min, followed by drying at 80 °C until a constant weight was obtained. The dried samples were ground and pulverized, and 0.5 g of powder was weighed and placed in a 250 mL conical flask, then digested with a mixture of HNO_3_:HClO_4_ (5:1 *v*/*v*). Element measurement was performed using a flame photometer (AP1302, Oupu Analytical Instruments Co., Ltd., Ningbo, China) [48].

### 4.6. Real-Time Fluorescence Quantification

The total RNA of cucumber leaves was extracted according to the instructions of the Tiangen plant total RNA extraction kit (DP432, Tiangen, Beijing, China), the reverse transcription kit was used to synthesize cDNA (KR118, Tiangen, China), and the SuperReal fluorescent quantitative premix reagent enhanced version (SYBR Green, FP205, Tiangen, China) was used for quantitative analysis. The primer information is shown in Table 1.

### 4.7. Statistical Analysis

Microsoft Office Excel 2010 was used to collate the data. Data analysis was used for Duncan’s multiple range test using SPSS software (version 25.0; IBM SPSS, Chicago, IL, USA). The level of significance test was *p* < 0.05. GraphPad prism 8 was used for graphing, and data were shown as the mean ± s tandard error (SE) of three independent tests.

## 5. Conclusions

**Figure 10:**
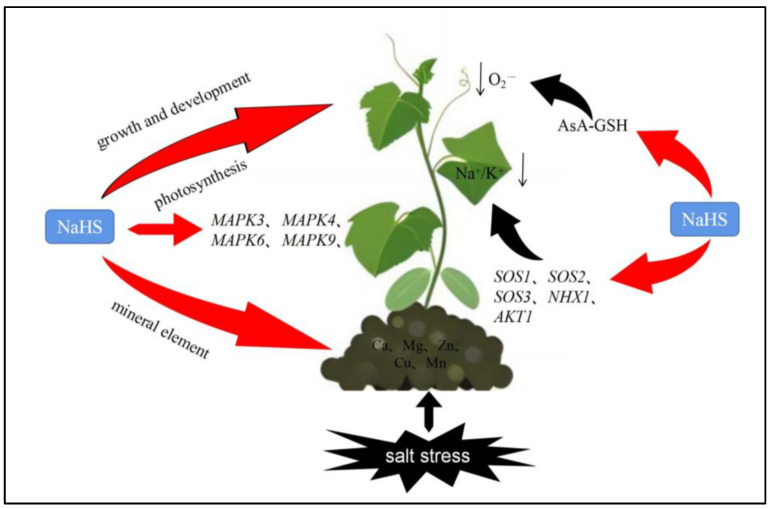
Schematic diagram for the effect of NaHS on salt stress. This figure shows that H_2_S alleviates salt stress and enhances salt resistance of cucumber through different ways. The red arrow indicates promotion, and the black arrow indicates inhibition.

In conclusion, the above results suggested that exogenous spraying of NaHS mitigated salt damage and maintained the growth of cucumber seedlings by improving photosynthesis and enhancing the AsA–GSH cycle, improving mineral ion uptake and reducing the Na^+^/K^+^ ratio. In addition, H_2_S was also found to alleviate salt stress and enhance salt resistance through the SOS pathway and MAPK pathway. A potential schematic showed the multiple pathways through which H_2_S improves salt tolerance in cucumber (Figure 10). This experiment explores the role of exogenous NaHS spraying in alleviating salt stress, providing reference for addressing the problem of soil salinization in facilities. At the same time, the interaction between H_2_S and other signaling molecules and the modification of H_2_S will be the direction of future research on alleviating salt stress.

## Figures and Tables

**Table 1:** Primer Information.

Gene Name	Forward Primer	Reverse Primer
*Actin*	GCCCTCCCTCATGCCATTCT	TCGGCAGTGGTGGTGAACAT
*SOS1*	CGGTAGCATGGTTGATTTTCG	GATTCGACCGGCTATGAGATG
*SOS2*	TGTGGAACCCCTGCTTATGTC	CGCACGACCAAATATCAGCTT
*SOS3*	CAAGGAAGAGTGGCGAAACC	TGGGAACGTGGTCGTGATATC
*MPK3*	GTCCTCCGATTATGCCTATTGG	TCTTAACCGCAACCATTTCGT
*MPK4*	AATCGACGCCAAAAGGACAT	CTCTTTTTGGCGGCCTAATG
*MPK6*	CCGTGCACCAGAGCTCTTACT	CAAGGGCTTCCGATCCATTA
*MPK9*	CGTGCTCCCGAACTTTGTG	TTCCAGTAAGCATTTCCGCA
*NHX1*	TGCTTTTGCCACCCTTTCA	TTCCAACCAGAACCAATCCC
*AKT1*	CTGTTCGTACAAAGCGATTG	TCCAACAAAACTCCTTCCAT

## Data Availability

Not applicable.

## Abbreviations

H_2_S	hydrogen sulfide
NaHS	sodium hydrosulfide
O_2_^·−^	superoxide anion
AsA	ascorbic acid
GSH	glutathione
GSSG	oxidized glutathione
DHA	oxidized ascorbic acid
SOS	salt overly sensitive
HKT	high-affinity potassium transporter
NHX	Na^+^/H^+^ antiporters
AKT	Arabidopsis-like potassium transporter
MAPK	mitogen-activated protein kinase
L-CD	L-cysteine desulfhydrase
NBT	Nitroblue tetrazolium
